# Duration of Untreated Eating Disorder and Prodrome in Young People: Characteristics and Relationship to Early Outcomes

**DOI:** 10.1111/eip.70164

**Published:** 2026-03-08

**Authors:** Jessica McClelland, Amelia Austin, Michaela Flynn, Victoria A. Mountford, Mima Simic, Ulrike Schmidt

**Affiliations:** ^1^ South London and Maudsley NHS Foundation Trust London UK; ^2^ Centre for Research in Eating and Weight Disorders King's College London, Institute of Psychiatry, Psychology and Neuroscience London UK; ^3^ Sage Clinics Dubai UAE

**Keywords:** anorexia nervosa, bulimia nervosa, duration of untreated eating disorder, eating disorders, prodrome

## Abstract

**Aims:**

Eating disorders (EDs) are serious illnesses and a better understanding of initial symptom phases could inform early intervention. This study aims to evaluate duration of untreated eating disorder (DUED) and duration/characteristics of the preceding prodrome period in young people diagnosed with an ED.

**Methods:**

Fifty‐four young people (28 adolescents aged 13–17 and 26 emerging adults aged 18–25) with recent onset EDs (< 3 years) completed a retrospective onset interview and life chart. DUED, prodrome duration and prodrome characteristics (ED‐specific and more broad psychiatric symptoms) were compared according to developmental stage and diagnosis. The relationships between DUED and prodrome duration with early outcomes were evaluated.

**Results:**

Emerging adults had a longer DUED than adolescents. This group also had a longer prodrome but only when broad psychiatric symptoms (i.e., not ED‐specific) were considered. Those with bulimia‐type EDs had a longer ED‐specific and broad psychiatric prodrome compared to those with anorexia‐type EDs. There were no significant developmental stage or diagnosis‐related differences in symptom severity during prodrome. In those who were underweight at presentation to services, a shorter broad prodrome duration was associated with greater weight gain after 3 months of treatment.

**Conclusions:**

When both ED and broad psychiatric symptoms are considered, DUED and prodromal symptoms of EDs differ according to developmental stage and diagnosis. Duration of prodrome may also impact early outcomes but further research is needed.

## 1 Introduction

Eating disorders (EDs) are characterised by disturbed eating and weight/shape concerns. They affect approximately 8% of women and 2% of men in their lifetime (Galmiche et al. 2019). Around 75% of people who develop an ED do so between the ages of 14 and 25 (Solmi et al. 2022), thus EDs typically arise during adolescence or emerging adulthood, a crucial stage of biopsychosocial development. EDs are associated with significant physical and psychosocial burden, psychiatric comorbidity, disability and mortality (Schmidt et al. 2025). EDs are conceptualised as developing from early risk and prodromal states into full‐threshold illness. Clinical staging models describe progression from early/mild illness to severe and enduring forms, with stage informing prognosis and treatment intensity (Treasure et al. 2015; Hyam et al. unpublished manuscript).

Prompt intervention is associated with improved chances for recovery (Ambwani et al. 2020; Treasure et al. 2015), therefore, services that treat early stage EDs are crucial. Such early intervention services have begun to emerge in EDs (Schmidt et al. 2016) and have reported superior clinical outcomes compared to usual treatment (Austin, Flynn, Shearer, et al. 2022; Fukutomi et al. 2020; McClelland et al. 2018; Richards et al. 2022). These services aim to reduce the duration of untreated ED (DUED), defined as the duration of time between illness onset and specialist ED treatment. Our review found that average DUED ranged from 2.5 to 6 years across the most common EDs and differed according to diagnosis (Austin et al. 2020). Moreover, DUED appears to be associated with age, meaning that younger individuals have a shorter DUED (Austin et al. 2020; Chew et al. 2021). Specifically, Weigel et al. (2014) found that compared to adolescents (under 18 years), emerging adults (aged 18–25 years) had a longer DUED of around 6 months. Importantly, predictors of outcome in early intervention have started to be explored, including baseline characteristics and early change trajectories (Allen et al. 2025; Austin, Flynn, Richards, et al. 2021).

Along with improved understanding of DUED, there is a need to know more about the earliest illness phase of EDs. This is known as ‘prodrome’ and relates to the period immediately preceding ED onset (Le Grange and Loeb 2007; Treasure et al. 2015). This period has been extensively researched in other psychiatric disorders, especially psychosis (Woodberry et al. 2016). Studies report on the duration, characteristics and predictive ability of prodromal psychosis (Barajas et al. 2019; Renwick et al. 2015; Valmaggia et al. 2015), leading to the development of robust prodrome assessment methods (e.g., Singh et al. 2005) and clinical services devoted to these prodromal ‘high‐risk’ stages of illness (Fusar‐Poli et al. 2013, 2016).

In comparison, little research exists on prodrome stages of ED, in part due to difficulties defining this (Treasure et al. 2015). As outlined by Stice et al. (2010) a *prodrome* is one or more symptoms which indicates and predates future ED onset. Raffi et al. (2000) assessed a range of symptoms 6 months prior to bulimia nervosa (BN) onset, finding that affective symptoms and low self‐esteem were the most common prodrome symptoms. In our evaluation of an early intervention ED service an average prodrome duration (including ED symptoms only) of 23.2 months was reported (Brown et al. 2018). Interestingly Del Barrio et al. (2022) found that young people with EDs presented more frequently to health services during ED prodrome, in relation to digestive, psychological, gynaecological and weight‐loss symptoms. More recently, compensatory weight‐control behaviours, lower weight status and the over‐evaluation of weight/shape were found to be the first prodromal symptoms of anorexia nervosa (AN). The same group identified prodromal binge eating and additional cognitive symptoms (e.g., feeling fat, fear of weight gain) to be associated with the onset of BN and BED (Yamamiya et al. 2023; Stice et al. 2021). Our latest study in young adults found that neither DUED nor duration of ED prodrome predicted clinical outcomes (e.g., weight, ED psychopathology and behaviours; Austin, Flynn, Richards, et al. 2025).

No standardised method of measuring prodromes in EDs exists. Given the high rates of psychiatric comorbidity in EDs (e.g., mood disorders ~54%, anxiety disorders ~62%; Hambleton et al. 2022) there is some ambiguity as to whether the prodrome is limited to the emergence of ED symptoms (*ED‐specific prodrome*) or includes broader psychiatric disorder‐related symptoms (*total prodrome*) (McClelland et al. 2020). This study aimed to gain a better understanding of DUED and the prodrome (both ED‐specific and total) in EDs. We compare the characteristics of DUED and prodromes between different developmental stages and diagnostic groups. We also explore the relationship between DUED and prodrome to early treatment outcomes. We hypothesised that:

- DUED and prodrome duration would be shorter in adolescents compared to emerging adults. Prodrome symptoms were expected to be less severe in adolescents compared to emerging adults.
- Shorter DUED and prodrome would be associated with better early treatment outcomes (e.g., improved psychopathology, weight gain).

## 2 Methods

Ethical approval for the project was given by the National Research Ethics Service Committee London (ref: 16/LO/1882).

### 2.1 Participants

Adolescents (aged 13–17) and emerging adults (aged 18–25) recently diagnosed with an ED as defined by the DSM‐5 (American Psychiatric Association 2013) were recruited (between 2017 and 2019) from the child and adolescent and adult ED services at the same hospital in South London, UK. Consent from the participant and/or a parent was obtained before enrolment in the study. Exclusion criteria were: age below/above 13–25 years, an ED duration > 3 years, no primary ED diagnosis, a severe physical/mental comorbidity requiring priority treatment, a severe learning disability or immediate inpatient admission.

### 2.2 Measures

*Onset interview*: An extended version of the onset interview and life chart we used previously was administered (Brown et al. 2018). This assessed the previous 5 years (rather than 3 years), in an attempt to capture both DUED and prodrome. Central to the interview is the *life chart*, which uses ‘anchor points’ (e.g., birthdays, starting university, etc.) to help orientate the young person to the timing of symptoms. The interview and life chart were completed by three of the authors, with extensive experience in these measures (via their involvement in FREED; authors J.M., A.A., M.F.). The completion of the interview and life chart was facilitated by researchers using an adapted version (including symptom onset, severity and progression) of the *Eating Disorder Diagnostic Scale (EDDS)* to assess the severity, frequency and duration of ED‐related psychological, behavioural and physical symptoms (Stice et al. 2000).

The *broad psychiatric symptom screener*, a novel interview schedule, captured the timing, duration and severity of other psychiatric disorder‐related symptoms/diagnoses. Information gathered from the adapted EDDS and the broad psychiatric symptom screener was used to collaboratively complete the 5‐year retrospective life chart with the young person in relation to the severity and impact of symptoms. After the interview, prodrome characteristics (onset, severity, distress, number of comorbid diagnoses), duration of ED‐specific prodrome, duration of total prodrome and DUED were calculated (see Box 1 for details). Further information on all materials can be found in the Supporting Information Material.

BOX 1 **Definitions of outcomes measured by the onset interview and life chart.**

**Duration of untreated eating disorder (DUED)**: The length of time between ED onset (as defined by DSM‐5 criteria) and the start of specialist, evidence‐based treatment.

**ED‐specific prodrome onset**: Date of first ED‐specific symptom (e.g. feeling fat, fear weight gain, influence weight/shape, excessive exercise, binge, purge or weight loss) at any rating greater than zero.

**Total prodrome onset**: Date of first psychiatric symptom, ED‐specific or otherwise (e.g., related to depression, anxiety, obsessive compulsive disorder [OCD], body dysmorphic disorder [BDD], substance abuse disorder [SUD]) at any rating greater than zero.

**ED onset**: Date symptoms met ED diagnosis as defined by DSM‐5.

**Duration of ED‐specific prodrome**: The length of time (months) between date of first ED‐specific symptom (e.g., feeling fat, fear weight gain, influence weight/shape, excessive exercise, binge, purge or weight loss) at any rating greater than zero and date of ED onset (as defined by DSM‐5 criteria).

**Duration of total prodrome**: The length of time (months) between the date of first psychiatric symptom, ED‐specific OR otherwise (e.g., related to depression, anxiety, OCD, BDD, SUD), at any rating greater than zero and the date of ED onset (as defined by DSM‐5 criteria).

**Symptom severity during prodrome**: Average rating across symptoms (ED psychological: 0 ‘not at all’ to 6 ‘extremely’; ED behavioural: 0 to 7 days/14 times per week; other psychiatric disorder related: 0 ‘not at all’ to 3 ‘nearly every day’) during prodrome.

**Number of other psychiatric disorder‐related diagnoses during prodrome**: Total number of other psychiatric‐disorder related diagnoses (e.g., depression, anxiety, OCD, BDD, SUD) during prodrome.

**Distress related to and impact of difficulties during prodrome**: Ratings of level of distress/impact (0 ‘no distress/impact’ to 10 ‘significant distress/impact’) of the ED‐specific and other psychiatric disorder‐related symptoms during prodrome.

*Eating Disorder Examination Questionnaire (EDE‐Q)—Version 6*. The EDE‐Q evaluated ED behaviours and cognitions in the past 28 days (Fairburn and Beglin 2008). High scores indicate more severe symptoms, with a proposed clinical cut‐off score of ≥ 2.8 (Mond et al. 2008).

*Demographic and clinical characteristics* (e.g., age, ethnicity) were collected via research interview and from clinical records. Service information including date of initial clinical assessment, and early treatment outcomes (weight and EDE‐*Q* score) at 3 and 6 months after treatment start were extracted from clinical records.

### 2.3 Procedures

In the child and adolescent ED service, clinicians introduced the study to young people and their families early in treatment, whilst in the adult ED service, participants were those that took part in a larger study of a novel early intervention service (FREED‐Up; Allen et al. 2020; Flynn et al. 2021). Study information was provided, and consent was obtained before administration of the onset interview.

### 2.4 Analyses

Statistical analyses were performed using IBM SPSS software (Version 25). Normality and other relevant assumptions of variables were assessed (via Kolmogorov–Smirnov and Levene's test statistics) and as most data were non‐normally distributed, non‐parametric tests and statistics were used. All tests were two tailed and with *α* = 0.05 significance.

Mann–Whitney and Pearson's chi‐squared tests were used to compare demographic and clinical characteristics. The effect sizes for these are *r* and Cramer's *V* respectively. Their interpretation of magnitude is 0.1–0.3 small, 0.3–0.5 medium, and > 0.5 large.

Kaplan–Meier survival analysis was used to compare differences (defined by hazard functions) in DUED, duration of ED‐specific and total prodrome according to developmental stage and diagnosis. The hazard function represents the probability of remaining in the dependent variable (e.g., DUED, prodrome) at any given time. Diagnostic subgroups were broadly split into anorexia nervosa (AN; including atypical AN), and BN or binge eating disorder (BED).

## 3 Results

### 3.1 Sample Characteristics

A total of 54 young people (predominantly female, 94%), 28 adolescents and 26 emerging adults, participated in the study and were included in analyses. Table 1 presents demographic and baseline clinical information for the entire sample and for subgroups. Comparing the adolescent and emerging adult groups, there were significant differences in ethnicity (higher proportion of white young adults; *χ* ^2^[2, *n* = 54] = 9.59, *p* = 0.01. *V =* 0.42) and, for those with AN, BMI (lower in adolescents; *U* = 62.50, *p* = 0.01, *r* = 0.43). There were no other significant differences between the developmental stage groups across demographic and clinical characteristics at the time of research interview. However, there was a significant difference in the duration of time between initial clinical assessment and the time of research interview (*U* = 90.50, *p* < 0.001, *r* = 0.66), with a larger gap in time (in months) for adolescents (Mdn = 3, Min = 0, Max = 25) compared to emerging adults (Mdn = 0, Min = 0, Max = 3). As expected, diagnostic comparison (i.e., AN vs. BN/BED) showed significant differences in weight, indicated by BMI (*U* = 57, *p <* 0.001, *r* = 0.63) and percentage weight for height (*U* = 9, *p <* 0.001, *r* = 0.50) at clinical assessment. There were no other significant differences between diagnostic groups.

**TABLE 1:** Demographic information at clinical assessment.

	All (*n* = 54)	Adolescents (*n* = 28)	Emerging adults (*n* = 26)	All AN/AAN (*n* = 32)	All BN/BED (*n* = 16)	Other EDs (*n* = 6)
Age	17 (13, 26)	15 (13, 17)	19 (17, 26)	17 (13, 24)	16 (14, 26)	
Gender	Female (*n* = 51)	Female (*n* = 28)	Female (*n* = 23)	Female (*n* = 30)	Female (*n* = 16)	Female (*n* = 5)
	Male (*n* = 3)	Male (*n* = 0)	Male (*n* = 3)	Male (*n* = 2)	Male (*n* = 0)	Male (*n* = 1)
Ethnicity	White (*n* = 38)	White (*n* = 17)	White (*n* = 21)	White (*n* = 24)	White (*n* = 11)	White (*n* = 3)
	Asian (*n* = 3)	Asian (*n* = 0)	Asian (*n* = 3)	Asian (*n* = 2)	Asian (*n* = 1)	Asian (*n* = 0)
	Other (*n* = 13)	Other (*n* = 11)	Other (*n* = 2)	Other (*n* = 6)	Other (*n* = 4)	Other (*n* = 3)
ED diagnosis	AN (*n* = 32)	AN (*n* = 17)	AN (*n* = 15)	n/a	n/a	ARFID (*n* = 2)
	BN/BED (*n* = 16)	BN/BED (*n* = 8)	BN/BED (*n* = 8)			
	OSFED (*n* = 4)	OSFED (*n* = 1)	OSFED (*n* = 3)			OSFED (*n* = 4)
	ARFID (*n* = 2)	ARFID (*n* = 2)				
BMI						
AN/AAN	17.90 (14.38, 25.30)	17.50 (14.38, 25.30)	18.70 (14.70, 25.00)	17.90 (14.38, 25.30)	n/a	
BN/BED	23.00 (18.40, 30.90)	22.35 (18.40, 30.90)	23.00 (18.90, 24.70)	n/a	23.00 (18.40, 30.90)	
Other EDs	20.75 (17.67, 24.50)	18.79 (17.67, 24.50)	21.20 (20.30, 23.10)	n/a	n/a	18.79 (17.76, 24.50)
% W4H						
AN/AAN	n/a	84.40 (17.32, 133.06)	n/a	84.40 (17.32, 133.06)	n/a	
BN/BED		119.73 (93.80, 146.00)		n/a	113.76 (93.80, 146)	92.31 (84.26, 117)
Other EDs		92.31 (84.26, 117.00)		n/a	n/a	

*Note:* Median and range reported.

Abbreviations: %W4H, percentage weight for height; AN, anorexia nervosa; BED, binge eating disorder; BN, bulimia nervosa; ED, eating disorder.

### 3.2 Duration of Untreated Eating Disorder

**FIGURE 1:**
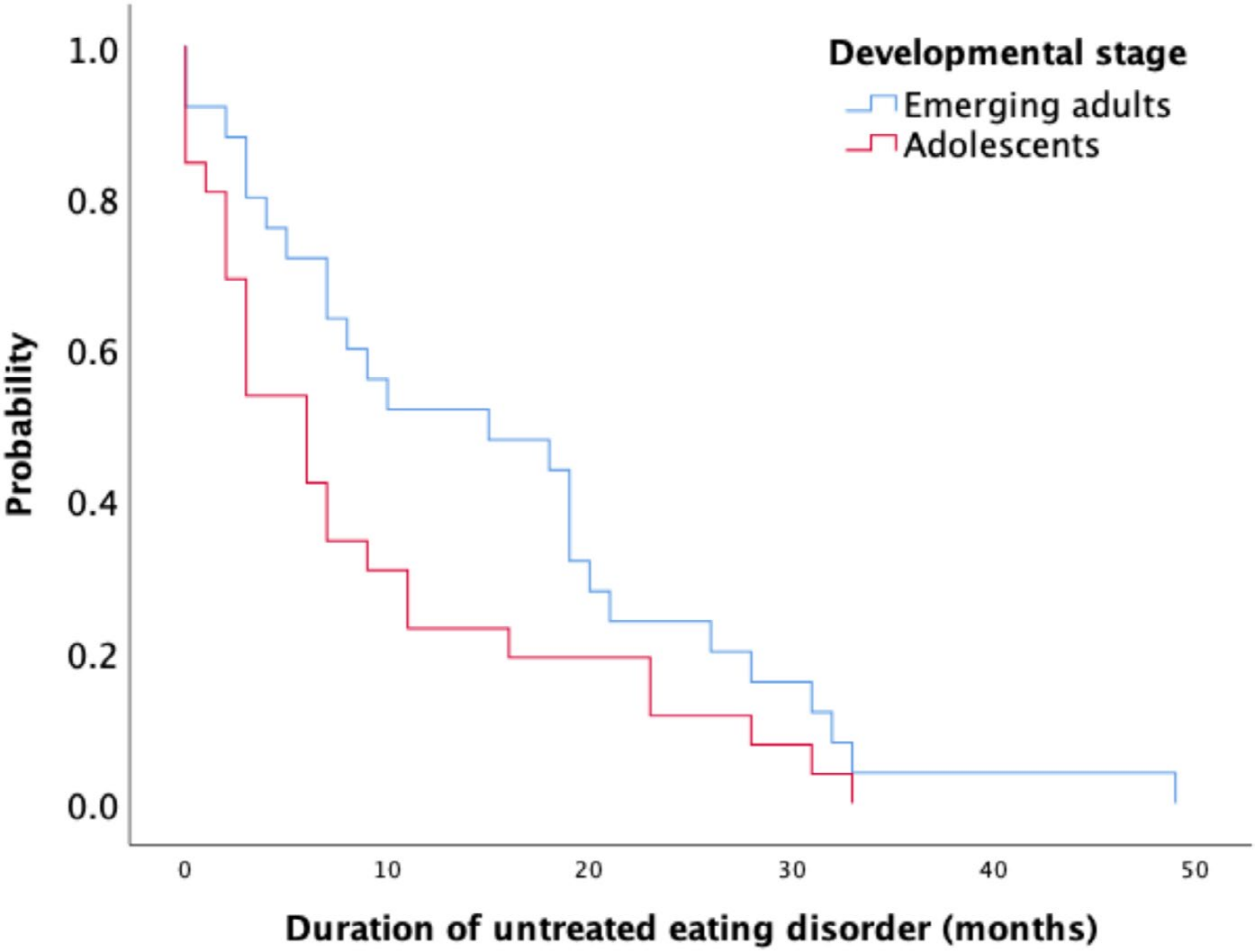
Duration of untreated eating disorder (DUED) according to developmental stage.

As represented in Figure 1 there was a trend towards a significant difference in the hazard functions of DUED (*χ* ^2^[1, *n* = 51] = 3.01, *p* = 0.08) between adolescents (Mdn = 6 months) and emerging adults (Mdn = 15 months). There were no significant differences (*χ* ^2^[1, *n* = 45] = 0.44, *p* = 0.51) in the hazard functions of DUED between those with AN (Mdn = 7 months) and those with BN/BED (Mdn = 7 months).

### 3.3 Duration of Eating Disorder Specific Prodrome

**FIGURE 2:**
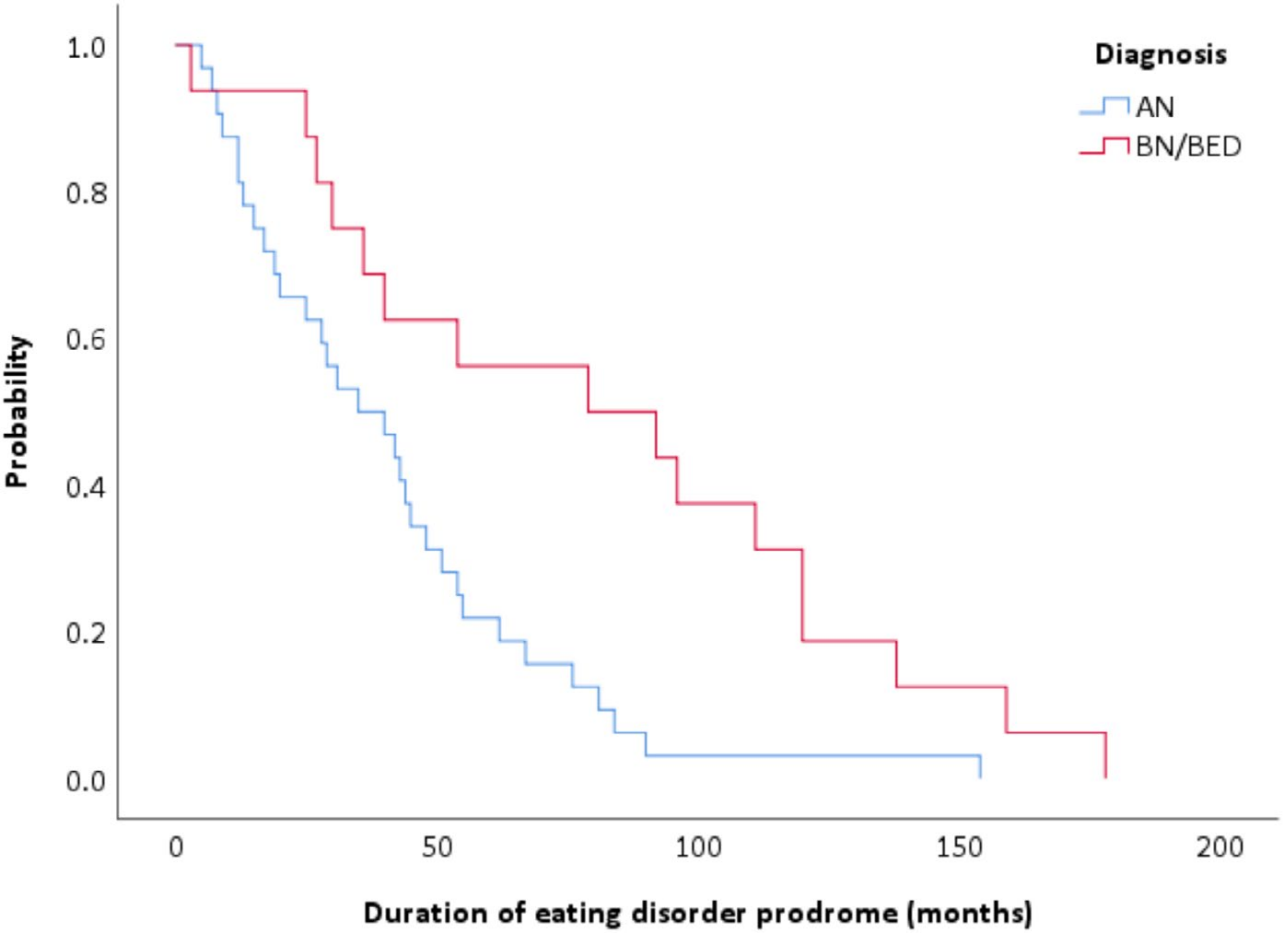
Duration of eating disorder specific prodrome according to diagnosis.

There was no significant difference (*χ* ^2^[1, *n* = 54] = 1.58, *p* = 0.21) in the hazard functions between the two developmental groups (adolescent Mdn = 36 months; emerging adults Mdn = 40 months). However, there was a significant difference (*χ* ^2^[1, *n* = 48] = 8.92, *p* < 0.01) in the hazard functions between those with AN (Mdn = 35 months) versus those with BN/BED (Mdn = 79 months; see Figure 2).

### 3.4 Duration of Total Prodrome

**FIGURE 3:**
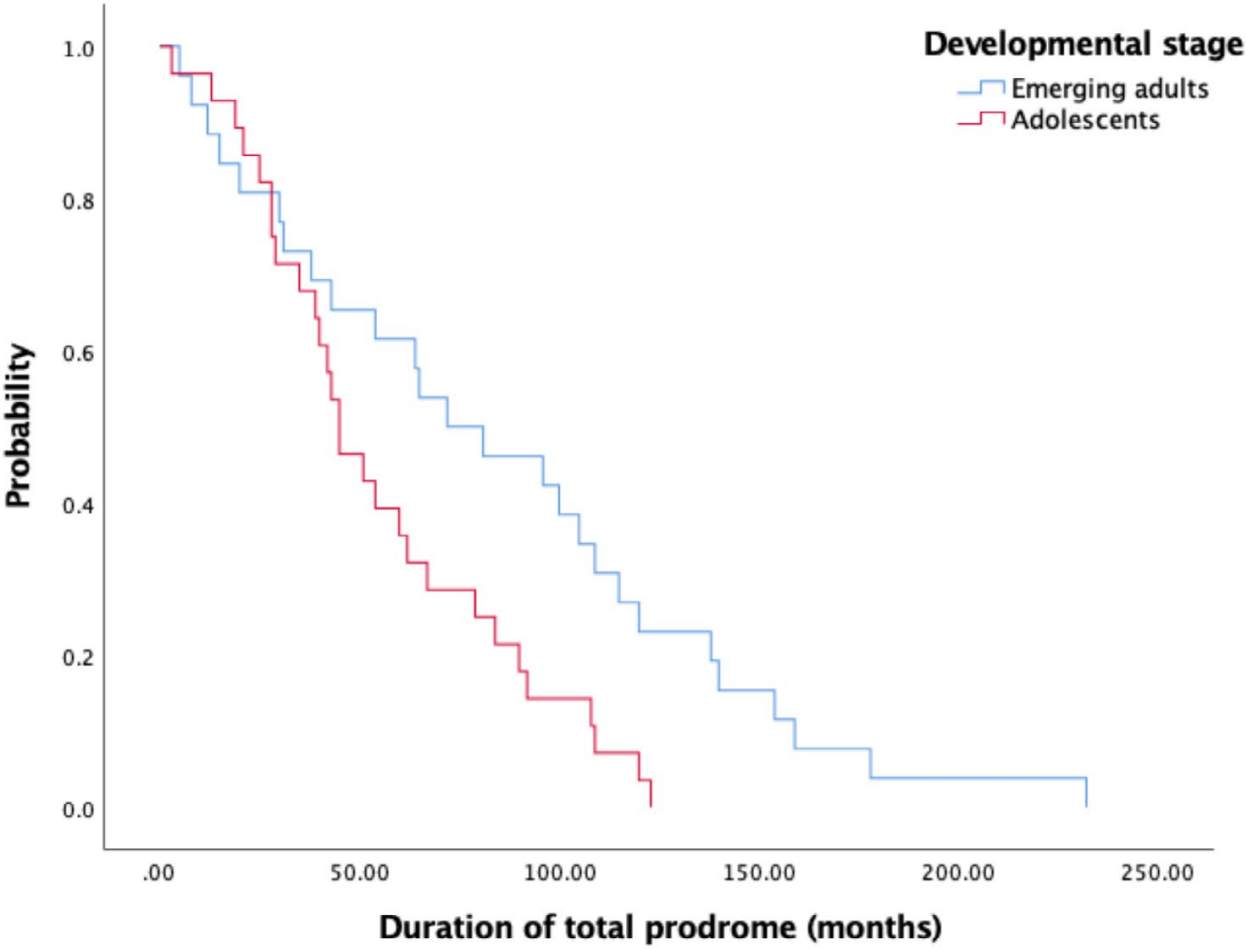
Duration of total prodrome according to developmental stage.

**FIGURE 4:**
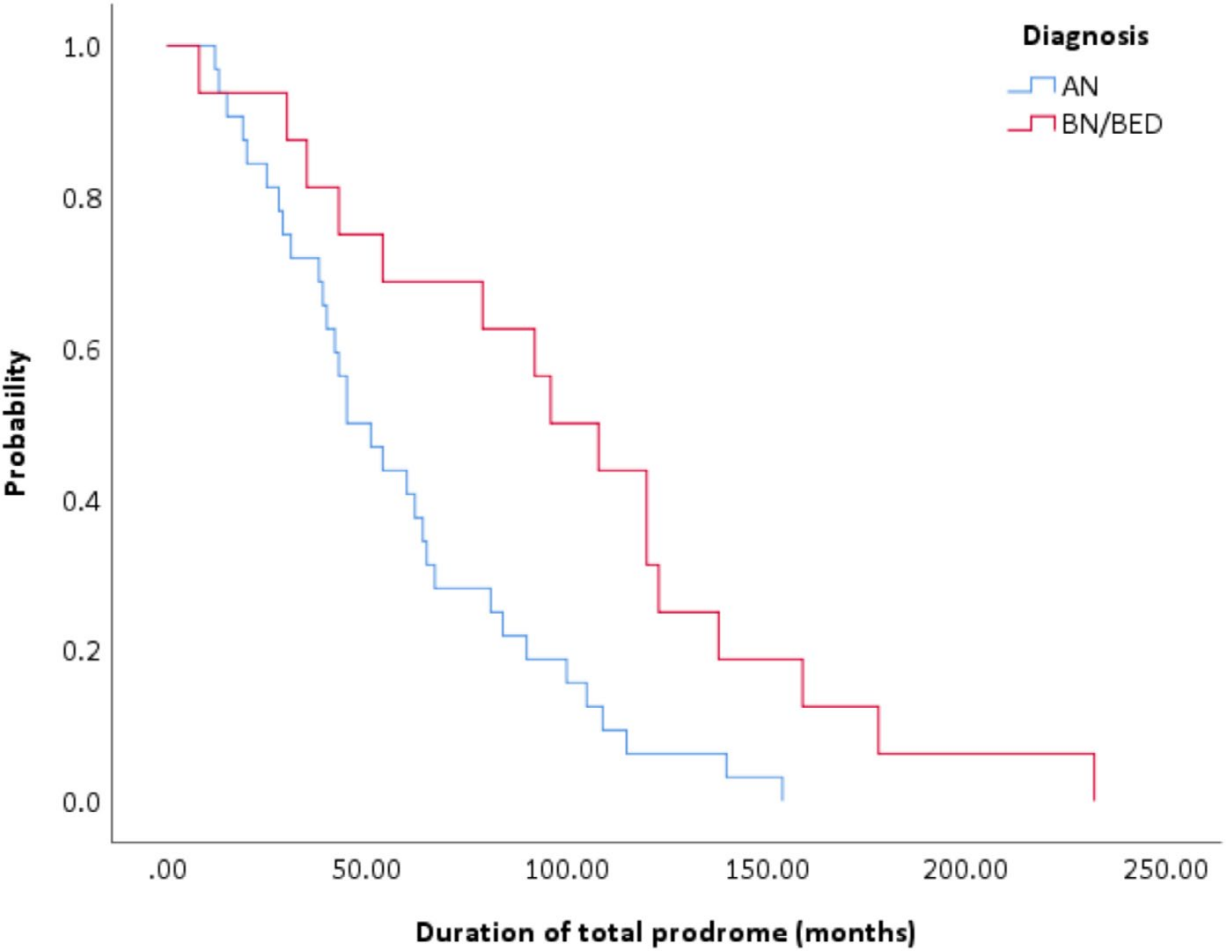
Duration of total prodrome according to diagnosis.

As demonstrated in Figure 3, there was a significant difference (*χ* ^2^[1, *n* = 54] = 6.02, *p* = 0.01) in the hazard functions between adolescents (Mdn = 45 months) and emerging adults (Mdn = 72 months) and between those with AN (Mdn = 45 months) versus those with BN/BED (Mdn = 96 months; *χ* ^2^(1, *n* = 48) = 7.83, *p* < 0.01; see Figure 4).

### 3.5 Prodrome Characteristics

Prodrome characteristics are presented in Table 2 for the entire sample and for age/diagnosis subgroups. Adolescents had a significantly earlier age of ED‐specific prodrome onset (*U* = 196.00, *p* < 0.01, *r* = 0.40) and total prodromal onset (*U* = 219.50, *p* = 0.03, *r* = 0.30) compared to emerging adults. There were no other significant differences between subgroups for any other prodrome characteristics, however there was a trend for individuals with BN/BED to have an earlier age of ED‐specific prodrome onset (*U* = 173, *p* = 0.07, *r* = 0.26) and increased distress during the prodrome (*U* = 93, *p* = 0.07, *r* = 0.31) compared to those with AN.

**TABLE 2:** Characteristics of prodrome period. Median and range reported.

		Developmental stage			Diagnosis		
	All (*n* = 54)	Adolescents (*n* = 28)	Emerging adults (*n* = 26)	*p*	AN/AAN (*n* = 32)	BN and BED (*n* = 16)	*p*
Age of ED prodromal onset (years)	12 (3, 23)	11 (3, 17)	13.50 (3, 23)	0.01	12 (8, 19)	9.50 (3, 21)	0.07
Age of total prodrome onset (years)	12 (2, 23)	12 (2, 16)	15.50 (4, 23)	0.03	12 (4, 23)	12 (2, 23)	0.98
ED psychological symptom severity (0–6)	4 (2, 5.7)	4 (2, 5.7)	4 (2.2, 5.75)	0.81	4 (2, 5.7)	4.1 (2.1, 5.7)	0.15
ED binge/purge symptom severity (0–14)	3.5 (1, 14)	3 (1, 7.5)	4 (1.3, 14)	0.15	3 (1, 14)	4 (1, 5.7)	0.48
ED fasting/restriction symptom severity (0–7)	5.25 (1, 7)	5.25 (1, 7)	6 (2, 7)	0.14	5.5 (1, 7)	5 (2, 7)	0.93
Number of other psychiatric disorder‐related diagnoses	2 (0, 7)	2 (0, 6)	2 (0, 7)	0.80	2 (0, 7)	1 (0, 4)	0.26
Severity of broad psychiatric disorder‐related symptoms (0–3)	2.2 (1, 3)	2 (1.67, 3)	2.4 (1, 3)	0.32	2 (1, 3)	2.4 (1.7, 3)	0.10
Distress from difficulties (0–10)	8 (0, 10)	8 (0, 10)	8 (2, 10)	0.58	7 (0, 10)	8.5 (3, 10)	0.07
Impact of difficulties (0–10)	6 (0, 10)	6 (2, 10)	7 (0, 10)	0.23	5 (0, 10)	7 (4, 10)	0.50

Abbreviations: AN, anorexia nervosa; BED, binge eating disorder; BN, bulimia nervosa; ED, eating disorder.

### 3.6 Relationship to Early Outcomes

There was no significant relationship between DUED or prodrome (both ED‐specific or total) with ED psychopathology at 3 or 6 months after starting treatment (*n* = 22). In those who were underweight (BMI ≤ 18.5 kg/m^2^) at initial clinical assessment there was no significant relationship between DUED and weight gain at 3 months (*n* = 21) or 6 months (*n* = 16) after starting treatment. However, there was a significant correlation between duration of total prodrome (*r*_s_ = −0.52, *p* = 0.02, *n* = 21) with weight gain 3 months after starting treatment (i.e., shorter prodrome related to greater weight gain). At 6 months after starting treatment, there was no significant relationship between either ED‐specific or total prodrome duration and weight gain (*n* = 16). Of note, participants were typically still in early stages of treatment at the time of analyses, therefore data were sparse at the 3‐ and 6‐month time points.

## 4 Discussion

This study explored characteristics of early illness stages of EDs in young people according to developmental stage and diagnosis. Our first hypothesis, that DUED would be shorter in adolescents compared to emerging adults was supported, which fits with previous evidence (Austin et al. 2020; Weigel et al. 2014). This finding may be influenced by a number of factors. The differing demands of developmental stages (e.g., increased independence, transitions and responsibilities in young adults), related differences in help‐seeking behaviours (Copeland et al. 2015; Potterton et al. 2020; Nicula et al. 2022), i.e., increased parental input in accessing support and the mandated access and waiting times for adolescents compared to adults (i.e., treatment must start within 28 days [NHS England 2015, 2019]). Surprisingly, no diagnostic related differences in DUED were found, unlike our review which indicated that internationally DUED is longest in non‐underweight EDs (e.g., ~67 months in BED, ~53 months in BN and ~30 months in AN; Austin et al. 2020). This could be explained by our small, uneven sample sizes across diagnostic categories.

Our second hypothesis, that prodrome duration would be shorter and symptoms less severe in adolescents compared to emerging adults, was partially supported. While there was no significant difference in the duration of ED‐specific prodrome, when broad psychiatric disorder‐related symptoms were considered, adolescents had a shorter total prodrome compared to emerging adults. This suggests emerging adults may have a longer period of broad psychiatric symptoms prior to ED onset, which is important to consider when designing developmentally tailored prevention and early intervention strategies. In terms of symptom severity, whilst emerging adults described some heightened severity during the prodrome compared to adolescents (which is in line with previous findings, e.g., Fisher 2003; Hudson et al. 2007; Swanson et al. 2011), these differences were not significant.

Comparison of the duration and severity of prodrome by diagnosis (across all ages) revealed that those with BN/BED had a longer duration of ED‐specific and total prodrome and greater levels of distress during prodrome compared to those with AN, although this finding is limited by uneven sample sizes. These are in line with our previous findings of longer DUED in BN/BED (Austin et al. 2020) and could be explained by weight stigma, the tendency for EDs in non‐underweight individuals to often go unnoticed and their severity typically dismissed. Raffi et al. (2000) did not make any comparisons in relation to prodromal EDs, our findings are consistent with their reports of affective symptomatology during prodromal stages of BN.

Our third hypothesis was also partly supported, with shorter total prodrome associated with increased weight gain 3 months after starting treatment. This is similar to our findings elsewhere (Austin, Flynn, Shearer, et al. 2022; McClelland et al. 2018), and highlights the impact of early intervention, with consideration of broad psychiatric symptoms, on quick initial weight restoration. We did not find a relationship between prodrome duration and any other early outcomes, possibly in part due to our small sample size at these follow up time points.

### 4.1 Strengths and Limitations

To our knowledge, this is the first study to thoroughly and broadly investigate how early stages of EDs may differ in terms of duration and severity according to developmental stage and diagnoses. However, our methodology had some limitations. Firstly, the normalisation of diet culture is likely to have impacted the accuracy of historical accounts of ED symptoms. Relatedly, the retrospective nature of this study will have led to recall errors and biases, limiting validity and reliability. The simple Likert scales used to assess symptom severity may have impeded the ability to detect changes over time. Moreover, whilst the interview was designed to capture a broad range of symptoms, there were several important omissions, including symptoms of other developmentally relevant disorders (e.g., ARFID, ADHD) and other possible prodromal features (e.g., early childhood eating difficulties, low self‐esteem; Del Barrio et al. 2022; McClelland et al. 2020). Finally, several findings were nearing significance, indicating that this study is likely underpowered. The small, predominantly female sample in this study limits the reliability and generalisability of the current findings.

### 4.2 Clinical/Policy Implications and Future Research Directions

The clinical differences we found between developmental groups (i.e., DUED and duration of total prodrome) supports recent calls for age and stage appropriate access to care for young people with AN (Herpertz‐Dahlmann et al. 2021). Given our findings that those with BN/BED have an extended prodrome, and a trend for increased distress, this call to arms needs to include all ED diagnostic categories, not just those of low weight. Specifically worrisome is the lengthy nature of the DUED and total prodrome for emerging adults compared to adolescents. As mentioned previously, this may be due to different patterns in help seeking between developmental groups. Interventions that aim to promote help seeking targeted to emerging adults are needed and are currently being developed (Grycuk et al. 2025). The differences in durations between developmental groups may also be impacted by unequal service provision. Internationally, services/care pathways for EDs are often split between child/adolescent services and adult services, even though the onset of these illnesses straddles adolescence and emerging adulthood (Treasure, Oyeleye, et al. 2021). This parallel service structure results in disparate care provision depending on arbitrary age cut‐offs. Possible solutions include establishing ED services which span the age range and the introduction of similar access time targets for all. Finally, clinical applications of improved prodrome measurement in EDs include better identification of ultra‐high‐risk individuals for early intervention and increased measurement accuracy of ED symptom duration. Future studies should aim to improve the interview process in ways previously mentioned before replicating. Following such research, the refinement of the lengthy interview into a simple screening tool would be useful for both research and clinical purposes, as has been seen in other disorders (Register‐Brown and Hong 2014; Van Meter et al. 2016).

### 4.3 Conclusions

Emerging adults had a longer DUED than adolescents, as well as a longer prodrome when broad psychiatric symptoms were considered. Those with BN/BED had a longer duration of both ED‐specific and broad prodrome compared to those with AN. Shorter prodrome duration of broad psychiatric symptoms was associated with early weight gain in treatment, highlighting the importance of broad early identification and intervention. These novel findings require replication. Future work must refine the assessment tools and evaluate the clinical implications of an improved understanding of DUED and ED prodromes. Such research could inform early detection and intervention strategies, for example by tailoring these to the unique needs of emerging adults and BN/BED presentations, ultimately interrupting the course of EDs becoming treatment resistant and life‐threatening for many young people.

## Funding

This work was supported by the Economic and Social Research Council (MR/W002418/1), Arts and Humanities Research Council (MR/W002418/1), Medical Research Council (MR/W002418/1) and National Institute for Health and Care Research (NIHR) Maudsley Biomedical Research Centre (BRC).

## Supporting information


**Data S1:** eip70164‐sup‐0001‐Supinfo.docx.

## Data Availability

The data that support the findings of this study are available on request from the corresponding author. The data are not publicly available due to privacy or ethical restrictions.
