# Supplementary material for: Duration of Untreated Eating Disorder and Prodrome in Young People: Characteristics and Relationship to Early Outcomes
Source: Early Interv Psychiatry. 2026 Mar 8;20(3):e70164. doi: 10.1111/eip.70164 (PMC12968482; doi:10.1111/eip.70164)
Supplement: Supplementary file 1 — Data S1: eip70164‐sup‐0001‐Supinfo.docx. [file EIP-20-0-s001.docx]

### Supplementary Methods

### 1.0 Measures:

### 1.1 Onset interview:

### An extended version of the retrospective onset interview, previously developed and piloted in EDs (Brown et al., 2018) was used. Central to the interview is the life chart, which uses ‘anchor points’ (e.g., birthdays, starting university, holidays, etc.) to help orientate the young person in time and inform estimates regarding the onset symptoms. For the purpose of this study, the life chart used in Brown et al. (2018) was improved in two ways – a) it evaluated five (rather than three) years prior to initial clinical assessment and b) it included other psychiatric disorder-related symptoms (i.e., beyond EDs). Using the individual ‘anchor points’, information gathered via the Eating Disorder Diagnostic Scale (EDDS; Stice, Telch & Rizvi, 2000) and other psychiatric disorder-related symptom screener (both detailed below) was mapped onto the chart collaboratively with the young person. Additionally, the overall distress and impact on functional areas of life (e.g., school/work, relationships) caused by ED and other psychiatric symptoms throughout this five-year period was rated from 0 ‘no impact/distress’ to 10 ‘significant impact/distress’.

##### **1.2 Eating Disorder Diagnostic Scale (EDDS), adapted.** The version of the EDDS that was previously adapted and piloted to determine the prodrome (including a question for each symptom regarding when it first started and the severity/frequency rating at this time) and ED onset was administered (Brown et al., 2018). The original version of the EDDS includes a range of questions assessing the severity, frequency and duration of psychological (e.g. feeling fat, influence of weight/shape on self-evaluation; rated from 0 ‘not at all’ to 6 ‘extremely’), behavioural (e.g. binge eating, fasting, restriction rated from 0-7 times per week; compensatory behaviours rated from 0-14 times per week), and physical symptoms (e.g. weight changes) which enabled DSM-5 diagnostic criteria for an ED to be established. Information gathered during the adapted EDDS was used to collaboratively complete the retrospective life chart with the young person. Following the research assessment, the completed onset interview and the life chart was used to calculate prodromal and ED onset, as defined by the DSM-5.

##### **1.3 Broad psychiatric disorder-related symptom screener.** We developed an interview schedule which aimed to determine other psychiatric-disorder related symptoms/diagnoses during prodrome. Symptoms that related to the DSM-5 diagnostic criteria of depression, panic disorder, social anxiety disorder, agoraphobia, specific phobia, illness anxiety disorder, generalised anxiety disorder (GAD), obsessive compulsive disorder (OCD), body dysmorphic disorder (BDD) and substance use disorder (SUD), were rated on a scale of 0 ‘not at all’ to 3 ‘nearly every day’. Information gathered during this interview regarding timing, duration and severity of other psychiatric disorder-related symptoms over the past five years was used to collaboratively complete the retrospective life chart with the young person and later used to calculate symptom severity and diagnoses. From the interview, the outcomes defined in Box 1 can be calculated.
